# Reply to Erre et al: translating cardio-rheumatology into practice: a multi-level pilot network

**DOI:** 10.1093/ehjcvp/pvag061

**Published:** 2026-09-16

**Authors:** Anne Grete Semb, Eirik Ikdahl, Anne Kerola

**Affiliations:** Department of Rheumatology, Division of Research and Innovation, Diakonhjemmet Hospital, Diakonveien 12, Oslo 0370, Norway; Department of Rheumatology, Division of Research and Innovation, Diakonhjemmet Hospital, Diakonveien 12, Oslo 0370, Norway; Department of Internal Medicine, Päijät-Häme Central Hospital, Wellbeing Services County of Päijät-Häme, Keskussairaalankatu 7, Lahti 15850, Finland; Institute for Molecular Medicine Finland, HiLIFE, University of Helsinki, Tukholmankatu 8, Helsinki 00250, Finland

Dear Editor,

We thank Prof. Erre, Dr. Sanna and the Italian CORDIS Study Group for their thoughtful comment on our PharmaPulse manuscript, *Why Cardio-Rheumatology?* They highlight the importance of Cardio-Rheumatology as an emerging subspecialty and emphasize the need to translate current knowledge into practical implementation of cardiovascular (CV) disease prevention in patients with rheumatic diseases. We fully agree with these perspectives.

The Preventive Cardio-Rheuma Clinic in Oslo was established in 2008 by a cardiologist. Over nearly two decades of clinical practice, we have identified several barriers to effective CV prevention in patients with inflammatory rheumatic diseases.

Awareness of the increased CV risk associated with rheumatic diseases remains insufficient among both healthcare professionals and patients. Improving health literacy in this area is therefore essential and can be achieved through several complementary strategies.

At the patient level, awareness initiatives delivered through patient organizations can promote patient empowerment and encourage preventive care. One example is the Norwegian Rheumatism Association’s (NRF) ‘Check It’ campaign, which focuses on the increased CV risk associated with rheumatic diseases.^1^ Through the NRF website, patients are encouraged to contact their general practitioner (GP) for assessment of blood pressure, lipid profile, and glucose levels. Patients receive a pamphlet containing a ‘Check It card’ on which these values can be recorded during their GP visit. These materials are also distributed through community pharmacies, thereby facilitating discussions between patients and their GPs regarding CV risk assessment.

Improving knowledge among healthcare professionals is equally important. Educational initiatives, including webinars, the European Association of Preventive Cardiology preventive cardiology course, and targeted dissemination through national primary care networks, may help increase awareness of CV risk prediction and prevention in patients with rheumatic diseases.

The European Alliance of Associations for Rheumatology recommends that rheumatologists ensure that patients with rheumatic diseases undergo CV risk assessment.^2^ In Norway, this recommendation has been implemented through the Norwegian Collaboration on Atherosclerosis in Patients with Rheumatic Joint Diseases (the NOCAR project), a nationwide quality improvement project established in 2014 involving 11 rheumatology clinics. The additional workload in routine rheumatology practice was minimal: lipid measurements were added to standard laboratory testing, patients self-reported CV risk factors, and blood pressure measurements were incorporated into the routine outpatient assessment alongside the joint examination. CV risk was calculated using the European SCORE algorithm and evaluated by the treating rheumatologist. Patients identified as having high or very high CV risk were referred to their primary care physician for initiation of preventive treatment, establishing practical shared care between rheumatology and primary care.^3^

Achieving guideline-recommended CV prevention targets remains challenging in the general population and may be even more important in individuals at high CV risk. Data from the Survey of Risk Factors in Rheumatoid Arthritis (SURF-RA) demonstrated that patients with both rheumatoid arthritis (RA) and diabetes mellitus received CV preventive medication more frequently than patients with RA alone. Lipid targets were also achieved more often in patients with both conditions, while blood pressure control was generally satisfactory across both groups.^4^ These findings suggest that the structured CV prevention strategies successfully implemented in diabetes care may serve as a model for improving CV prevention in RA. For instance, integrating disease-specific risk multipliers directly into electronic health record tools (such as SCORE-2) via automated prompts can help reduce therapeutic inertia in general practice.

Dedicated Cardio-Rheuma clinics are unlikely to be feasible on a large scale because of resource constraints. However, multidisciplinary collaborations within existing tertiary referral centres are both realistic and increasingly common. Such clinics have been established at several academic institutions, including the Cleveland Clinic, Mayo Clinic, NYU Langone, Yale University, Stanford University, Brigham and Women’s Hospital, Women’s College Hospital in Toronto, and at Diakonhjemmet Hospital in Oslo. Rather than operating in isolation, such centres can serve as regional coordination hubs that host and support virtual multidisciplinary boards for surrounding clinical networks, as proposed by CORDIS.

The organizational structure of these clinics depends on local healthcare systems and available resources. Cardio-Rheumatology services may be integrated within rheumatology departments, organized as dedicated joint clinics, or based within cardiology departments. Our experience from the Preventive Cardio-Rheuma clinic in Oslo, embedded within a rheumatology outpatient service, using rheumatology principles of tight disease control and treat-to-target, demonstrated achievement of recommended lipid targets in approximately 86% of referred patients after a median of only two consultations.^5^ These findings demonstrate that guideline-recommended lipid management is achievable in patients with rheumatic joint diseases and underscore the value of close collaboration between rheumatologists and cardiologists.

Improving CV prevention in patients with rheumatic diseases requires coordinated efforts at multiple levels, including increased patient awareness, education of healthcare professionals, systematic CV risk assessment within rheumatology practice, and effective collaboration between rheumatologists, cardiologists, and primary care physicians. While dedicated cardio-rheumatology clinics may not be feasible in all healthcare settings, the principles underpinning these multidisciplinary models can be broadly implemented to improve CV outcomes in this high-risk population.

Kind regards

## Data Availability

This is a respone to a comment on a Pharmapulse manuscript. There is no data available.
